# Retrospective Review of Medical and Surgical Science in the United States of America, during the Last 30 Years

**Published:** 1820-12-01

**Authors:** 


### *Retrospective Review of the Improvement of Medical and Surgical Science in the United States, during the last thirty years.**

"A RETROSPECT of the improvements in medical science, in our own country, during the last thirty years, cannot, it is presumed, be considered uninteresting. To look back occasionally, on the scene over which we have passed—to contemplate the progress we have made —is indeed not only interesting, but highly useful.

"In such a retrospect, however, we cannot hope, always, to trace with precision the silent and gradually progressive steps of scientific improvement; for conspicuous facts and remarkable discoveries are not the only sources from which improvement in science derives its impulse. With the gradual, but imperceptible progress of the human mind towards perfection, science moves slowly forward in the path of improvement, without our being able to point out, with definiteness, whence its momentum is derived. Facts and observations, also, scarcely appreciable in their separate and isolated state, acquire importance, and become obvious in their influence, only when, in process of time, they are brought into association and comparison with others. Nor can we pretend always to indicate with clearness the full value of what may be deemed the more important facts or discoveries; for, although we may see the bright lights occasionally set up in the dominions of our science, yet we cannot follow those infinite irradiations which they send into the obscurities of nature, and which contribute, though almost insensibly, to develop her secrets.

"Such are the general difficulties naturally connected with our task. But there are also others to be encountered, of a peculiar character, in giving an account of the improvements of American science. For such is the constant intercourse between this country and Europe—so rapidly is the light of improvement reflected from one country to the other—and so readily are the sparks elicited in the one, caught and nursed into light in the other, that it is often extremely difficult, and sometimes impossible, to say, with positive assurance, to which the credit of original improvement belongs.

"Medicine has been cultivated with much zeal and success in America, within the period of our retrospect. A free, active, and enterprising spirit of enquiry, and an independence of the doctrines and authorities of European writers, characterizes, in an especial manner, the medical mind of our country. 'This hemisphere,' it has been truly said, 'is the theatre on which the prejudices and errors

---

* *American Medical Recorder*, Vol. II. *October*, 1819. The English reader must bear in mind that it is the *American* Reviewer who speaks in this article.

of the European schools, in a great variety of instances, have been refuted and abandoned, and on which new principles in medicine have been proposed, ascertained, and completely established.'

" In noticing the progress of medical improvement in the United States, our view is necessarily directed, principally, to the University of Pennsylvania. For this institution may with propriety be denominated the *punctum saliens* of medical science in America. It is to the free and independent spirit of inquiry, which some of its eminent teachers have manifested, in the pursuit of medical science, as well as to the equally bold and unrestrained spirit of investigation, which for many years characterized the pupils of this institution, that we are to look for almost every thing that is valuable in the improvement of American medicine.

" Previous to about the year 1790, the doctrines and principles generally entertained by our physicians were European. The systems of Boerhaave and Cullen were almost universally adopted amongst us. In the remediate treatment of diseases, however, American physicians, although entertaining the pathological sentiments of European writers, early found themselves obliged to strike out new plans of cure, or to pursue the modes of treatment proposed in the books, with a boldness and energy corresponding with the more rude and vigorous features of our native diseases.

" Dr. Rush was the first physician who promulgated an *American* system of medicine. This system, which was at once bold, plausible, and novel in its views, forms an important epoch in American medicine. It gave a national character to the medical science of our country. ' It rejects,' to use its author's own words, ' the nosological arrangement of diseases, and places all its numerous forms in morbid excitement, induced by irritants acting upon previous debility. It rejects, likewise, all prescriptions for the names of diseases, and by directing their applications wholly to the forming and fluctuating state of diseases, and the system, derives from a few active medicines all the advantages which have been in vain expected from the numerous articles which compose European treatises upon the materia medica.'

" Whatever we may now think of this system, in all its parts, it cannot be denied that it embraces many important and profound views, which, before its promulgation, were but dimly seen, or altogether unknown. By it, we have been taught a more just and simple doctrine concerning the radical and general relation of diseases, as well as a more rational estimate of the importance of nosological arrangements. That the doctrines and opinions taught by this illustrious benefactor of our science have descended with him into the tomb—that they are the mere meteor lights of fancy, which have passed away with the mind from which they flashed, few, we presume, will allow, notwithstanding the sentence of oblivion pronounced against them by his biographer, Dr. Caldwell.

" Among the most important improvements which have been introduced into medicine in America, may be reckoned the more accurate and rational views entertained at the present day, concerning the origin and causes of epidemic diseases, as well as a more correct estimation of the effects and value of quarantine regulations.

"Drs. Rush,* Miller, Physick, Caldwell, Mitchell, and Pascalis, deserve to be particularly noticed, for having improved our notions concerning these subjects. The two former, especially, have thrown much light on the origin and character of the yellow fever of this country, and of epidemic and pestilential diseases in general. The monster contagion was in this country divested of his terrifying aspect, and his powers shown to be less dangerous and universal, than they were at one time supposed to be.

"Besides the improvement of our knowledge concerning the general character of diseases, and their intimate relations, many important diseases separately considered have been more thoroughly investigated, and their nature and treatment more correctly ascertained.

"The nature and treatment of dropsical complaints, are, at present, much better understood than they were formerly. Dr. Rush has the merit of having first advanced correct views and principles concerning the pathology and treatment of these diseases. He removed them from the class cachexia, and placed them, very justly, with febrile diseases. He showed, by a train of incontrovertible argument, that dropsy is a disease attended by morbid excitement, and preternatural action of the arterial system. This view of hydropic diseases deserves to be regarded as forming a very important improvement in modern pathology. The mode of treatment deducible from it, is at once more rational and successful, and the sentiments as well as the practice of our illustrious countryman, in relation to this disease, are daily becoming more prevalent, both in Europe and in America.—It may be remarked, however, that while these improved views are becoming more common in Europe, the sources, whence they are immediately derived, are not duly recognized. Dr.

---

"* In the *Dictionaire des Sciences Medicales*, *tome* xv. *page* 546, in the article, '*Fiévre Jaune, est elle contagieuse?*' written by Fournier and Vaidy, we meet with the following extraordinary paragraph: '*B. Rush avait d'abord cru que la maladie était contagieuse: il a soutenu, depuis* 1802, *une opinion contraire. Mais ce medecin a déclaré, en mourant, qu'il avait en cela cédé à des considérations particuliéres, et qu'il n'a jamais cessé de croire que la fiévre jaune est contagieuse. Il a désavoué à son heure suprême, tout ce qu'il avait écrit en faveur de la non-contagion. Nous tenons cette anecdote de témoins dignes de foi, parmi lesquels il suffit de nommer M. Moreau de Saint Méry.*'—'B. Rush believed at first that this disease is contagious. Since the year 1802 he advocated a contrary opinion. But he declared on his death-bed that he did so from private considerations, and that he had never ceased to believe in the contagiousness of yellow fever. He disavowed, in his last moments, all that he had ever written in favour of non-contagion. We derive this anecdote from undoubted testimony, amongst whom it is sufficient to mention M. Moreau Saint Méry.' *Credat Judæus!* This scandalous and ridiculous *falsehood* needs no formal refutation. Here in America we *know* it to be *untrue*, the testimony of M. de Saint Méry to the contrary notwithstanding.

Abercrombie* of Edinburgh, in an essay on the use of blood-letting in certain dropsical affections, proposes to prove, what no American physician doubts, 'that dropsy is often found existing in a state of the body directly the reverse of exhaustion, and even in immediate connexion with symptoms of an inflammatory nature;' in other words, he sets out to prove, what Dr. Rush had long before proved much more ably, that dropsy is a disease, generally, if not universally, attended with '*morbid excitement and preternatural action of the arterial system.*' The name of Rush is not once mentioned by Dr. Abercrombie, notwithstanding the pains he seems to have taken, in hunting up isolated facts and authorities, among the older writers, in support of the sentiments he delivers. In a review of Dr. Parry's elements of pathology, in the 58th number of the same Journal, the credit of removing dropsy from the class cachexia, is given to Dr. Blackall, without referring to Rush, to whom alone, this credit rightfully belongs—'We regard it,' says the reviewer, 'as no mean triumph of modern pathology, that dropsy is removed from the class cachexia, that it is no longer considered as the product of depraved solids, but a disorder of the sanguiferous system. The researches of Dr. Blackall, a few years ago, and existence of albumen in the urine in many cases of idiopathic dropsy, have opened the minds of the profession to a belief of its general alliance to inflammation.' The instances, however, of European writers, availing themselves, without due acknowledgments, of the suggestions and improvements of American physicians, are by no means few. We shall have occasion to notice several glaring ones, in the course of this paper. The following is another example: 'I have not used,' says Dr. Duncan, in an account of the late epidemic fever in Scotland, 'Cullen's distinctions of synochus and typhus, because, I do not believe that the distinction exists in nature. I have preferred distinguishing the cases by the epithets, *cephalic*, *pulmonic*, *gastric*, *enteritic*, *hepatic*, &c. from the principal organs affected.' This nomenclature was long ago proposed and adopted by Dr. Rush. Dr. Duncan does not give him credit for it.†

"Among the late general improvements of medical science, effected principally in this country, may be mentioned a more simple and efficacious treatment of pestilential diseases, and a more discriminating, rational, and decisive employment of the remediate articles, and especially of blood-letting in febrile diseases.

"Mania a potu, has lately been treated in a new and successful manner with emetics, by Dr. Jos. Klapp. From an experience suf-

---

* Edinburgh Medical and Surgical Journal, No. 55, 1818.

† We can pretty confidently assure the American Reviewer that the silence of British writers respecting American medical literature, is *not* a studied one. Transatlantic works have had no circulation in this country to speak of, and the interchange of Journals has hitherto been but very imperfect We hope we shall remove this cause of complaint in future —*English Edit.*

ficiently extensive, to warrant a comparative estimate, we cannot doubt of the superior efficacy of this mode of treatment. The usefulness of emetics in mania a potu, would seem to go considerably towards confirming the correctness of the gastric pathology of certain mental and cephalic diseases, a pathology which has of late gained much ground in Europe, and in some degree, in our own country. That the primary irritation of this class of disorders does often consist in *functional* or organic derangement of the chylopoetic viscera, there can be no doubt. In this country, these pathological views, are perhaps too much neglected. Dr. Chapman, however, with an enlightened discrimination has given due weight to the gastric pathology of mental and cephalic diseases, and has contributed much to the dissemination of these sentiments amongst us.

"The successful application of the tincture of guaiacum in dysmenorrhœa, as recommended and practised by Dr. Dewees, deserves to be noticed among the improvements of practical medicine. By the use of this remedy, the membrane formed on the internal surface of the uterus in this disorder, and which causes difficult and painful menstruation, as well as sterility in the married woman, is expelled, and its subsequent formation prevented, and the pain and barrenness which arose from its presence obviated.

"*Surgery* has received numerous and important improvements in the United States. It is practised here, perhaps, in a more perfect state than in any other country. 'The American surgeon,' says Dr. Dorsey, 'is, or ought to be, strictly impartial, and therefore adopts from all nations their respective improvements.' Whilst the value of the doctrines of adhesion are fully understood with us, which is not the case with the French, the improvements of French surgery, neglected by the English, receive here an equal attention.

"Dr. Physick has contributed largely to the improvement of surgery. Indeed there are few subjects in this department of the healing art, that have not received some favourable modification from him. What has been said of the poet, may with the utmost propriety be repeated of this gentleman, in relation to surgery, *nihil tetigit quod non ornavit.*

"In the modification and invention of surgical instruments, many valuable improvements have been made in America. Dr. Physick's improvement on the gorget is important. It consists in having the beak and blade formed of two separate pieces, and so constructed as to be readily and firmly united. By this contrivance the blade may be separated from the beak, and a perfectly keen edge given to that part of it which commences the incision, an advantage which cannot be gained when the beak and blade are inseparable. This is obviously an improvement of much value. For a particular description, see Dorsey's Surgery, vol. ii. page 175.

"The French mode of treating fractures has been generally adopted in this country, so far, at least, as relates to the use of permanent extension and counter-extension, for the purpose of keeping the extremities of the fractured bone in opposition. Important modifications, however, in the modes of keeping up extension and counter-extension in fractures, have been made in America.

" The long splint of Desault, used in fractures of the thigh, has received a useful modification from Dr. Physick. The improvement consists in increasing the length of the splint, so that the counter-extension is more in the direction of the thigh.

" In oblique fractures of the leg, a very convenient and effectual mode of applying permanent extension was contrived by the late Dr. James Hutchinson. This is an improvement of much value in the treatment of fractures of the leg. For a particular description of this splint, and its mode of application, see Dorsey's Elements of Surgery, vol. i. p. 178.

" An apparatus for applying permanent extension and counter-extension, in fracture of the thigh, has also been invented by Dr. Hartshorne. By this contrivance, the extension and counter-extension are made by a splint placed on the inside of the leg, reaching from the perinæum to about six inches below the foot. The upper extremity is cut out like the top-piece of a crutch, which is lined and stuffed with hair. This part, resting upon the perinæum and ischium, serves as a support to the counter-extending force. The lower extremity does not differ from that of Hutchinson's splint.*

" The angular splints, used by Dr. Physick in fracture of the os humeri, at or near the condyles, to prevent a deformity which is extremely apt to occur in these cases, is also an improvement deserving notice. Dr. Physick has ascertained that the same advantage may be more certainly obtained, by keeping the patient in bed, ' with the arm flexed at the elbow, and lying on its outside with the rectangular splints supported by a pillow.'†

" Surgeons have complained of the difficulty of turning a needle, when introduced into deep and narrow wounds for the purpose of taking up deep seated arteries. To obviate this difficulty, Dr. Physick has introduced the use of an instrument which completely answers the purpose. It consists in a *curved* forceps, by which the needle is held, and thus rendered perfectly manageable. Heister describes an instrument for holding needles in making sutures. Dr. Physick, however, was the first surgeon who proposed and used such an instrument, *for the purpose of taking up deep-seated arteries.* In the Eclectic Repertory, a modification of this mode of holding the needle in taking up deep seated arteries, is described by Drs. Parish, Hewson, and Hartshorne. These gentlemen have forgotten to mention Dr. Physick as the *first* surgeon who used such an instrument for this purpose. Dr. Physick's improvement was made in 1800.

" Blisters, when locally applied to a part in a state of mortification consequent to inflammation, will generally put an immediate stop

* Hartshorne's Translation of Boyer on the Bones, page 365.

† Dorsey's Elements of Surgery, vol. i page 159.

to its progress. This very important remedy was introduced into practice by Dr. Physick. He first employed it in 1803. The blister should be large enough to cover all the sound parts in contact with the diseased. 'I have witnessed its effects,' says Dr. Dorsey, 'in a variety of instances, and have no hesitatian in recommending it in preference to all other local remedies. After the first dressing of the blister, it will generally be found that the mortification has ceased to progress, and in a short time the separation of the sloughs commence.'

"Dr. Physick's mode of treating artificial anus is unquestionably one of the finest improvements of modern surgery. This mode of treatment consists in consolidating the sides of the intestines laterally, for a short distance below the artificial opening. To effect this union a ligature was passed by Dr. P. through the intestine, and suffered to remain a week, keeping its side in close contact. A portion of this consolidated partition between the two extremities of the intestine was afterwards removed by a cutting instrument, the fæces thus regaining their natural route, the external aperture was healed up without difficulty. This important improvement is claimed in France by Dupuytren;* and in Germany, where it is spoken of as a most valuable improvement, it is also given to this surgeon. Dr. Physick, however, performed this operation in January, 1809, successfully, long before Dupuytren's case occurred.†

---

* Anzeige einer operations weise zur heilung des *anus artificialis*, nebst bemerkungen von Dr. Resinger.

† Upon this subject, it gives us pleasure to be able to introduce the following letter from our friend, G. S. Pattison, Esq. late professor of anatomy, physiology, and surgery, in the Andersonian University, Glasgow.

"Dear Sir, *Pine Street*, *Aug.* 28, 1819.

"In compliance with your request, I now transmit you an account of the method adopted by M. Dupuytren of Paris, for the cure of artificial anus.

"In most cases of artificial anus, the superior and inferior portions of the wounded gut are connected with each other at the external wound: they, in fact, as it has been aptly said, lie along side of each other, like the tubes of a double-barelled gun. The rationale of cure proceeds upon this their locality. 'All that is required,' says the French surgeon, 'is to open a lateral communication between the different portions of the intestine; if this be done, it is evident that the fæculent contents will pass with greater facility through that opening, and descend, *per vias naturales*, than overcome the stricture of the wound, and be discharged externally.' The operation which this gentleman performs for the accomplishment of the cure, has two intentions. 1st. To make a direct opening of communication betwixt the superior and inferior portions of the intestine. 2dly. To guard against fæculent effusions into the abdomen. It is performed by introducing separately into the different portions of the gut, the blades of forceps, which have a considerable resemblance in shape to those which are used for the extraction of bullets. So soon as these have been united at the hinge, and it has been ascertained by the finger that the blades grasp the in-

" The application of blisters to the tract of an inflamed vein, is a practice of much value. This treatment was first introduced by Dr. Physick. 'A small plaister of simple cerate, spread on linen, is to be applied on the orifice, and over this a blister is to be laid large enough to cover the whole inflamed part, extending three or four inches from the orifice in every direction.'

" In cases of retention of urine, it is often impossible to introduce a catheter, although a bougie may be introduced without difficulty. A case of this kind having occurred to Dr. Physick in 1796, he fastened the point of a bougie upon the extremity of an elastic catheter, and thus very readily passed the instrument, which he had previously attempted in vain. 'For this purpose, a French catheter of the middle size is to be provided, and its point cut off, leaving a continued cylindrical canal through it. A piece of bougie plaister between two and three inches long is to be cut into a proper shape, forming a triangular piece, the base of which is about one inch and a quarter, and the height about three inches. When it is rolled up, as in making bougies, sufficiently to fill the cavity of the catheter, a slit half an inch long is to be made in its lower end, after which the part already rolled up is inserted into the catheter, and the other half is wrapped round its outer side, and fastened by tying a cambric thread neatly round it, In order to secure still more effectually the bougie point from slipping off, and to extract it, in case this ac-

---

side of the intestines immediately interior to the external opening, they are pressed so forcibly together as to destroy the vitality of all the parts embraced betwixt them. The forceps are now withdrawn, and Nature finishes the cure. The manner in which she accomplishes this will be easily understood. The pressure having destroyed the life of all the substance placed betwixt the blades of the forceps, it must necessarily be separated from the living parts: it must slough off: and as a certain degree of inflammation is required before this can happen, the parts surrounding, from their being covered with serous membrane, are glued together by coagulable lymph, and effusion prevented.

" With all other European surgeons, I believed, until my arrival in the United States, that this method of cure, which I consider one of the most philosophical and beautiful discoveries in modern surgery, was first introduced into practice by M. Dupuytren, of the Hotel Dieu. Indeed, I have in a work on the surgical anatomy of the trunk, which I have been for some time preparing for the press, so considered it, and given the due meed of praise to him, who I then considered its author. I have since learned to whom the discovery belongs, and I can assure you, that it will be a pleasing task for me to correct what I have written, and to publish an account of Dr. Physick's operation, which is essentially the same as the one performed by the French surgeon, and which, from its having been performed many years before in a public hospital, and continued to be taught session after session in a medical university, gives unquestionably all the honour of this great improvement in surgery to the HUNTER of America.

" I remain, dear sir, your's most truly,

" GRANVILLE SHARP PATTISON.

" To DR. EBERLE."

cident should happen, a strong thread is passed through the bougie, and fastened to the outer extremity of the catheter.'* This is very justly considered as one of the greatest improvements which the catheter has received in modern times.

" In false joints from fracture, Dr. Physick has introduced the practice of passing a seton through the diameter of the limb, between the two fragments of the bone, in order to inflame their extremities, and thereby to produce a reunion of the fractured bone. There is some dispute relative to the origin of this important practical improvement. Boyer, Roux, and a few other French authors, have by an ambiguous notice of this practice, conveyed, or evidently wished to convey, an impression that the merit of first introducieg this improvement into surgery is due to Mr. Percy. Boyer and Roux, who quote from Laroche, state, that 'Percy performed this operation two years before Dr. Physick's operation was *known* in France.' This does not, however, prove, though it insinuates, that Percy was the first who performed this operation. For, to say that an operation was performed by one surgeon, two years before he *knew* that another surgeon had performed a similar operation, does certainly not establish the priority of invention to the former. Both Boyer and Roux, moreover, express themselves with so much ambiguity on this subject, as to render it evident, that they themselves did not view Percy as preferring a just claim to the merit of introducing this valuable improvement. 'On pourroit, *peut-être*, réclamer,' says Roux, 'la prioritie de l'invention en faveur de M. Percy.' But Laroche, from whom Boyer and Roux take their account of this operation, gives the credit of first using it to Dr. Physick. He states, also, that Percy did not perform this operation with the express purpose of producing a reunion of the fractured extremities, but simply to facilitate the discharge of dead bone; and he adds, that having seen setons used after gun-shot fractures, to favour the escape of dead pieces of bone, he is astonished that he or his master never took the hint of applying this practice to the cure of artificial joints.

" In cases of retention of urine from stricture, where every attempt to introduce a catheter fails, surgeons generally recommend puncturing the bladder. To avoid this formidable operation. Dr. Physick contrived an operation, which he has repeatedly performed with success. It consists in perforating the stricture by an instrument consisting of a curved canula, or catheter, concealing a lancet capable of being protruded when necessary. 'The operation is to be performed, by introducing the instrument as far as it will go, and then the lancet is to be protruded. In some cases the obstruction is situated beyond the bend of the urethra: and in these cases, in order to guard against all danger of wounding any other parts except those intended, the handle of the instrument is to be depressed as low as possible; and when it is pushed onward, it will be found to have divided the stricture, and urine will generally escape through

---

* Dorsey's Surgery, vol. ii. p. 162.

the canula. The lancet is immediately to be retracted, and the urine evacuated. A catheter must afterwards be introduced, and left in the bladder until the new passage heals up. This very important operation has been repeatedly performed by Dr. Physick, and has never been followed by any unpleasant consequences.'*

"Dr. Physick's armed bistoury, for the operation of fistula in ano, is also an improvement of considerable value. 'It combines, in a great measure, the advantages of the blunt and sharp pointed bistouries, and possesses some advantages over both.'†

"In the reduction of dislocation of the thigh, Dr. Physick has also contrived a very effective mode of making extension and counter-extension. For a particular account of Dr. Physick's mode of applying the extending and counter-extending forces in dislocations of the thigh, see Dorsey's Surgery, vol. i. p. 260.

"The use of copious bleeding, in facilitating the reduction of luxations, though originally proposed by Dr. A. Monro, was first put into practice in our country by Dr. Physick. This eminent surgeon has also directed the attention of surgeons more particularly to making the counter-extension against the acromion process in the reduction of dislocations of the shoulder joint.

"In consequence of having remarked that strips of adhesive plaister applied over ulcers were soon dissolved in the pus discharged from them, Dr. Physick suggested, many years ago, the use of animal ligatures, for the purpose of taking up arteries. 'For as it is now sufficiently ascertained that all the processes requisite for the obliteration of a blood-vessel secured by a ligature are completed in a very short space of time, probably in two or three days, it follows, that if the ligature applied be made of materials capable of securing the vessel during this space of time, and liable to decomposition and solution in the animal fluids afterwards,' much advantage would be gained. Dr. Physick proposed the use of leather for this purpose; and in an experiment made in 1814, with a buck-skin ligature, applied to the large artery of a horse, it was found to afford all the advantages for which it was suggested. 'It restrained the bleeding, and was discharged in a liquid state in two or three days.'

"Dr. Physick's mode of treating *morbus coxarius* deserves to be mentioned in this place. He advises the application of a curved splint to the hip, in order to secure the perfect rest of young patients, which, without such a contrivance, it is almost impossible to do, and which is of the utmost importance in the cure of this disease. Active and long purging, together with a low diet, make up the rest of the treatment.

"It has long been a question of much difficulty, with surgeons, why inflammations so readily take place in cavities that are laid open to the admission of the external air. Hunter ascribed it to the '*stimulus of imperfection*;' Abernethy, 'to the frequent renewal

---

* Dorsey's Surgery, vol. ii. p. 163.

† Ibid. vol ii. p. 188.

and long continued application of air to a surface unaccustomed to it;' and John Bell, 'to the length of the incision,' &c. This question received at last a satisfactory solution by the late Dr. James Cocke of Maryland, in his inaugural dissertation, published at Philadelphia, in 1804. This gentleman shewed, by a variety of well-devised and satisfactory experiments, 'that the inflammation which supervenes on the surfaces of wounded cavities is *the consequence of the change and diminution of temperature caused by the* admission of air into them. That the vessels are first debilitated by the abstraction of their natural heat, and that they afterwards take on an increased action and inflame.'*

"Dr. J. R. Barton has lately invented a very ingenious and valuable mode of bandaging the lower jaw, in the treatment of fractures and other injuries of this part. See 2d No. vol. ii. of this Journal.

"For preventing or arresting the progress of whitlow, Dr. Perkin of Philadelphia has introduced an excellent remedy. It consists in making an escar on the affected part by the early application of caustic.

"In *midwifery*, many valuable improvements, both theoretical and practical, have originated in this country. In cases of difficult parturition, depending on rigidity of the mouth of the uterus, Dr. Dewees has introduced a very important practice. He recommended many years ago, in such cases, copious bleeding, *usque ad deliquium animi*, and experience abundantly proves the utility of this practice.† So important was this treatment considered by the late professor Shippen, that he puplicly pronounced it, as forming an epoch in practical midwifery.

"When, in pregnancy, the placenta is situated over the mouth of the uterus, much difficulty and danger from hæmorrhage usually attends for some time previous to, and during parturition. In cases of this kind, the common practice is to break through the placenta and to deliver the child by the feet. Dr. Dewees has recommended, and used successfully, a mode of practice in such cases, which obviously possesses many advantages over the other methods commonly pursued. He directs the accoucheur to pass his hand up, between the placenta, membranes and the uterus to the top of this organ—there to rupture the membranes, and laying hold of the child's feet, to deliver it. The advantages of this mode of operating are first—Much less violence is done to the connexion of the placenta with the uterus, and thereby the risk of increased hæmorrhage diminished. 2. Much time is saved. 3. We arrive at the feet, and can command their descent with much more certainty. 4. We prevent an atony of the uterus by allowing the waters to escape gradually and at will. 5. It prevents the fœtus from being entangled in the placenta, and thus does away the inconvenience that would arise from the increase

* See Dorsey's Surgery, vol. i. page 91.

† An Essay on the means of lessening pain in certain cases of difficult parturition, by W. P. Dewees, 2d edition, 1819.

of bulk, as in the former method, the size of the placenta is added to that of the child. 6. It prevents the rude and sudden separation of the placenta from the uterus.

" There is no accident more to be deprecated in obstetrical practice than *inversio uteri.* Reduction is frequently impracticable, and in this case death generally follows speedily. When the inversion is incomplete, so that part of the uterus remains within the neck, whilst the fundus projects through it in an inverted state, it becomes strangulated, and its reduction is rendered impossible. In such cases, Dr. Dewees recommends, grasping the projecting tumour of the uterus firmly, and to bring it forwards, so as to complete the inversion. This operation he has practised with success, and with almost immediate relief to the patient.* The uterus afterwards contracts to a small size, and may, if it becomes inconvenient, be removed by ligature, as lately practised with success by Mr. Newnham of England.

"'That pain is not only unavoidable, but essentially necessary in parturition, is a doctrine which had never been contested by any one, we believe, until Dr. Dewees brought forward a contrary opinion, in an interesting paper, published in the 1st vol. of the Philadelphia Medical Museum. Lately, the same sentiments have been delivered by Dr. Power of England, in a book he has written on midwifery. This writer, though claiming to himself the merit of having developed new and important principles in midwifery, is entitled only to the minor praise of having *adopted* the sentiments of our countryman, Dr. Dewees.

" Ergot has lately been introduced into *regular* practice by Dr. Stearns of Albany. We are aware that this article was used in France and Germany more than a century ago. It was however not known in *regular* practice, until Dr. Stearns brought its virtues before the public ; and to him, therefore, rightfully belongs the merit of having first directed the attention of the medical public to the use of this valuable article in obstetrical practice.

" In a country like ours, extending through every variety of soil and climate, Nature, it is reasonable to suppose, has not neglected to bring forth many of her healing and balsamic plants. From the bosoms of our own forests, accordingly, have we already drawn a great variety of remediate articles, of the greatest utility in the cure of diseases.

" To no one are we more indebted for a knowledge of our indigenous Materia Medica, than to the late professor Barton. His zeal and industry in the cultivation of this department of medical science, his readiness to communicate to others the knowledge which he sedulously collected from every accessible source, concerning our Vegetable Materia Medica, and above all, the care which he constantly took to direct the attention of the medical students of the University of Pennsylvania to the investigation of the virtues of our native plants,

---

* See an Essay on the partial inversion of the uterus. By W. P. Dewees, in Coxe's Medical Museum, vol. vi. p. 21.

contributed in a remarkable manner to the increase of knowledge upon this subject.

"To notice, in detail, all our native medicinal plants, would be swelling this part of our retrospect beyond all proper bounds. We may enumerate the following, as amongst the most valuable of our lately discovered medicinal plants.

"Lobelia inflata heuchera Americana, rubus trivials, sanguinaria canadensis, geranium maculatum, orabanche virginiana, podopyllum peltatum, asclepias tuberosa, daucus carota, prunus verticilatus, cornus florida, ulmus fulva, diospyros virginiana, zanthoxylum fraxineum, magnolia glauca, prunus serotina, liriodendron tulipefera, rhus typhinum, rhus radicans, phytolacca decandria, coptis trifoliata, frasera walteri, baptesia tinctoria, cunilla mariana, eupatorium perfoliata, zanthorrhiza apiifolia, hydrastis canadenis, convolvulus panduratus, comptonia asplenifolia, euphorbia ipecacuanha, spirea trifoliata, gaultheria procumbens, triosteum perfoliatum, monarda punctata.*

"The polygala seneca, though long known as a valuable article of the Materia Medica, has had its virtues more accurately ascertained, within the period of our view. Dr. Archer, of Maryland, first noticed its excellent powers in the cure of cynanche trachealis, and Dr. Hartshorne first observed its emenagogue virtues.

"Cantharides also appear to possess very valuable emenagogue powers. For a full and accurate knowledge of the virtue of flies in promoting the menstrual discharge, we are indebted to Dr. Jos. Klapp. Several writers, we are aware, and amongst those Dr. Chapman in particular, have noticed these virtues of the flies, before Dr. Klapp's essay on this subject was published, but it does not appear that any one before him drew his conviction of their utility from actual and reiterated experience. See Dr. Klapp's Essay on Cantharides, in the 1st vol. of this journal.

"Physiology has not advanced as rapidly in this country, as most of the other branches of medicine. Practical medicine chiefly is the field in which the physicians of this country have particularly distinguished themselves. Physiology, nevertheless, has been cultivated with much care, and has received several important improvements amongst us. The subject of cuticular absorption in particular has been investigated with much attention and success by Drs. Klapp and Rousseau. By the experiments of these gentlemen, it seems to be demonstrated that no absorption can ever take place, from the cuticular surface of the human body.

"Dr. Mussey, however, performed a number of experiments, from which it would appear that madder does pass through the external surface into the circulation; but whether the passage of this substance through the cuticular surface was by absorption, or by percolation simply, is not as yet determined. Madder is certainly

* See Dr. Attbe's paper on this plant, in the present number of our Journal.

a very penetrating article. Substances, very little porous steeped in it, receive its colouring matter in so fixed a manner, as to make it almost impossible to remove it again. By bathing the body with it for some time, it creates a stinging and prickling pain in the skin. It is therefore very probable, that when madder does pass through the skin unto the circulation, it penetrates the cuticle mechanically, and coming in contact with the absorbents opening under the cuticle, is by them taken up and carried into the circulation.

"Dr. Rush's theory of the spleen deserves, we think, to be ranked among the modern improvements in physiology. According to his theory, the spleen serves as an important preservative organ in the animal economy. It constitutes a waste gate or reservoir for the torrent of blood excited into action by violent and excessive agitation of the blood vessels, whereby the more tender and vital organs are protected from the too violent effects of this force, and from dangerous congestions.

"Dr. James Johnson, in his valuable work on "*the liver, internal organs, and nervous system,*" says, 'When the balance of the circulation is broken, and the blood is determined from the surface upon the internal parts' (as in the cold stage of fevers) 'were it all to accumulate in the large vessels about the heart, and in the lungs, immediate death would be the consequence; but the local abstraction of so large a proportion of blood from *actual circulation*, by its quiescence in the *spleen* and portal circle, (where plethora is not so immediately detrimental) preserves the heart and lungs from being overpowered, till reaction restores the equilibrium between the surface and the interior. *From this view of the affair, the utility of the Spleen, as an organ of preservation, is no longer doubtful.*'

"It was for many ages believed, that the blood in certain diseases entered into putrefaction, or, at least, into its incipient stage. Hoffman and Cullen first opposed this doctrine, and rendered it extremely probable, by reasoning from general principles that this can never take place. What was thus rendered probable by reasoning upon the subject, Dr. Seybert demonstrated to be true, by a series of well-directed and conclusive experiments.*

"In anatomy, although cultivated with as much assiduity in this country as any other department of our science, we cannot boast of many discoveries. Anatomy is a field, in which the most patient and well directed inquiry can hope for little that is new. In Europe this science is cultivated with uncommon zeal, by men of the first grade of genius; and yet how seldom do they add any thing of importance to our knowledge of the human structure! After so many centuries of the most patient and acute examination of the human body, can it be wondered that so little, now, awaits the most prying researches of the anatomist?

---

* An attempt to disprove the putrefaction of the blood in living animals, by Dr. A. Seybert.

"The late Professor Wistar, shortly before his death, communicated to the Philosophical Society a paper, in which he proves, that the 'generally received account of the formation of the sphenoidal sinuses is not strictly correct.' He describes two small bones which have heretofore been considered as processes of the sphenoid bone, but which he demonstrates to be distinct from it.

"Dr. M'Clellan of Philadelphia has lately shewn, that 'the common idea respecting the extent of the pleuræ is incorrect. That they do not terminate at the first ribs, as is stated in all the books, but extend to some distance above them, and line the inner surface of the scaleni muscles.' Dr. M'C. has found the pleura to extend as high on each side as the thyroid cartilage. It is therefore obvious, that the pleura becomes involved, in all operations on the subclavian, and lower portion of the carotid arteries.

"We have thus endeavoured to notice such improvements in medicine, as have been made during the last thirty years. We cannot pretend to have given all the improvements with which medical science has been enriched in America. We have however given all we had a knowledge of; and if we have omitted some facts, and thereby not rendered justice to all the successful cultivators of our science, let it be ascribed to any thing but want of candour. *Suum cuique.*"

